# A Comprehensive Analysis of Citrus Tristeza Variants of Bhutan and Across the World

**DOI:** 10.3389/fmicb.2022.797463

**Published:** 2022-04-08

**Authors:** Dilip Kumar Ghosh, Amol Kokane, Sunil Kokane, Krishanu Mukherjee, Jigme Tenzin, Datta Surwase, Dhanshree Deshmukh, Mrugendra Gubyad, Kajal Kumar Biswas

**Affiliations:** ^1^Plant Virology Laboratory, ICAR-Central Citrus Research Institute, Nagpur, India; ^2^Whitney Laboratory for Marine Biosciences, University of Florida, St. Augustine, FL, United States; ^3^National Citrus Program, Department of Agriculture, Royal Government of Bhutan, Thimpu, Bhutan; ^4^Department of Plant Pathology, Indian Agricultural Research Institute, New Delhi, India

**Keywords:** citrus tristeza virus, genomic diversity, sequencing and phylogenetic analysis, RT-PCR, genomic regions

## Abstract

Mandarin orange is economically one of the most important fruit crops in Bhutan. However, in recent years, orange productivity has dropped due to severe infection of citrus tristeza virus (CTV) associated with the gradual decline of citrus orchards. Although the disease incidence has been reported, very limited information is available on genetic variability among the Bhutanese CTV variants. This study used reverse transcription PCR (RT-PCR) to detect CTV in collected field samples and recorded disease incidence up to 71.11% in Bhutan’s prominent citrus-growing regions. To elucidate the extent of genetic variabilities among the Bhutanese CTV variants, we targeted four independent genomic regions (5′ORF1a, p25, p23, and p18) and analyzed a total of 64 collected isolates. These genomic regions were amplified and sequenced for further comparative bioinformatics analysis. Comprehensive phylogenetic reconstructions of the GenBank deposited sequences, including the corresponding genomic locations from 53 whole-genome sequences, revealed unexpected and rich diversity among Bhutanese CTV variants. A resistant-breaking (RB) variant was also identified for the first time from the Asian subcontinent. Our analyses unambiguously identified five (T36, T3, T68, VT, and HA16-5) major, well-recognized CTV strains. Bhutanese CTV variants form two additional newly identified distinct clades with higher confidence, B1 and B2, named after Bhutan. The origin of each of these nine clades can be traced back to their root in the north-eastern region of India and Bhutan. Together, our study established a definitive framework for categorizing global CTV variants into their distinctive clades and provided novel insights into multiple genomic region-based genetic diversity assessments, including their pathogenicity status.

## Introduction

Tristeza, caused by the citrus tristeza virus (CTV), is a destructive disease affecting citrus plants. Over the past few decades, tristeza has damaged millions of productive citrus trees worldwide (Kokane et al., 2021c; Moreno et al., 2008; Ghosh et al., 2021). Bhutan is a small landlocked Himalayan country located between India and China, and is the likely place of origin of citrus (Wu et al., 2018). Diverse agro-climatic conditions are prevalent in the country, favoring the production of a wide range of horticultural crops, among which the citrus is the most important fruit crop (Joshi and Gurung, 2009). Mandarin (*Citrus reticulata* Blanco) is a widely grown citrus cultivar in 17 out of the 20 districts, constituting more than 95% of the total citrus grown in Bhutan (Joshi and Gurung, 2009; Dorji et al., 2016). The prominent mandarin-growing geographical regions are Tsirang, Dagana, Zhemgang, and Sarpang (Tipu and Fantazy, 2014; Ghosh et al., 2021). However, the citrus productivity in these regions is very low because of several factors, including infection of virus and virus-like pathogens. Among these pathogens, CTV is considered a major pathogen responsible for reducing the citrus yield and quality and decline of fruit-bearing citrus groves in Bhutan (Tipu and Fantazy, 2014).

Local transmission of the virus within a citrus groove is by aphid species, such as *Aphis gossypii* Glover, *Aphis* (Toxoptera) *citricidus* Kirkaldy, and *Aphis spiraecola* Patch, in a semi-persistent manner, whereas transmission into a new geographical area or country occurs through the movement of infected budwood during nursery propagation (Bar-Joseph et al., 1989; Marroquín et al., 2004; Herron et al., 2006). CTV is a phloem-limited virus having long flexuous filamentous particles of 2,000 × 11 nm in size and belongs to the genus *Closterovirus* under the family *Closteroviridae*. The single-stranded positive-sense RNA genome of ∼19.3 kb organized into 12 open reading frames (ORFs), which potentially encode 19 different proteins, and two untranslated regions (UTRs) located at the 5′ and the 3′ terminal (Manjunath et al., 1993; Pappu et al., 1993; Karasev et al., 1995; Biswas et al., 2018). The 5′ proximal ORF1a encodes a 349-kDa polyprotein that includes two cysteine papain proteins-like (PRO) domains, a methyltransferase-like (MT) and helicase-like (HEL) domains, and ORF1b encodes an RNA-dependent RNA polymerase (RdRp)-like domain. The 3′ genomic region of CTV consists of 10 ORFs (ORFs 2–11) that encode different proteins with diverse functions, namely, major (CP) and minor (CPm) coat proteins, p65 (a homolog of cellular HSP 70 proteins), and p61 that is required for virion assembly and movement along with the hydro-phobic p6 protein (Hilf et al., 1995; Satyanarayana et al., 2000; Dolja et al., 2006; Tatineni et al., 2008). Additionally, the p20 and p23 proteins function as suppressors of the host RNA silencing along with CP (Lu et al., 2004), and three genes (p33, p18, and p13) are needed for systemic infection and play a role in extending the virus–host range (Tatineni et al., 2008, 2011). There are numerous biological strains of CTV (Brlansky et al., 2003; Harper, 2013) that infect almost all commercial citrus species and induce a wide variety of symptoms including stem pitting, vein clearing, stunting, veins corking, chlorosis, leaf cupping, and slow or quick decline (Ghosh et al., 2008, 2009; Warghane et al., 2017a,b; Kokane A. et al., 2020). The expression of symptoms in field-infected plants depends on the type of host, virus strain, rootstock–scion combination, age of the citrus tree, and environmental conditions (Ghosh et al., 2018; Kokane A. et al., 2020). Biological indexing has been used as a classical method of detecting CTV for years, but it has certain limitations. Other techniques that have been used for CTV detection are enzyme-linked immunosorbent assay (ELISA) (Liu et al., 2016; Kokane S. B. et al., 2020), dot immunobinding assay (DIBA), and immunoelectron microscopy (IEM) with polyclonal or monoclonal antibodies (Ahlawat, 2012). However, the serological methods are not used extensively because they require a supply of good quality antisera.

Electron microscopy is another powerful technique, but it is very expensive, requires highly skilled personnel, and cannot distinguish viruses of similar size. However, the most sensitive virus detection methods that are being routinely used at present in different laboratories include reverse transcription polymerase chain reaction (RT-PCR) (Mehta et al., 1997), quantitative PCR (qPCR) (Ruiz-Ruiz et al., 2007; Kokane et al., 2021b), multiplex RT-PCR (Meena and Baranwal, 2016), and RT-LAMP (Warghane et al., 2017a; Kokane et al., 2021a). Recently, a rapid, sensitive, robust, reliable, and highly specific reverse transcription recombinase polymerase amplification technique coupled with a lateral flow immunochromatographic assay (CTV-RT-RPA-LFICA) has been developed for early detection of CTV (Ghosh et al., 2020). Apart from complete genome sequencing (Harper, 2013), the genetic diversity of CTV has also been determined based on different genomic regions by several researchers (Rubio et al., 2001; Martín et al., 2009). The phylogenetic analysis of CTV isolates using 5′ORF1a genomic region (Roy et al., 2005a; Atallah et al., 2016), coat protein region (p25) (Warghane et al., 2020), RNA binding protein gene (p23) (Flores et al., 2013; Kokane S. B. et al., 2020), and p18 gene has also been reported (Dawson et al., 2013). The major focus of our study was molecular detection, characterization, and determination of the genetic diversity of 64 CTV isolates based on sequence variations of four (5′ORF1a, p25, p23, and p18 gene) different genomic regions. The targeted regions span most of the CTV genome, and each region plays a specific role; for example, the highly variable region ORF1a encodes a polyprotein of MT, and HEL domains, p25 covers 95% coat protein, p23 plays a role as a major suppressor, and p18 is involved in systemic infection for extending the virus–host range.

Thus, we selected these four regions for a comprehensive analysis of CTV variants by comparing them with whole-genome sequences across the globe. By comparing the 64 CTV isolates along with the published and unpublished sequences deposited in the GenBank, we have shown that the Bhutanese isolates could be unambiguously classified under six (except for T30) of seven (RB, T36, T30, T3, T68, VT-B, and HA16-5) internationally recognized and two additional clades (B1 and B2) identified in this analysis. Our analysis provides a comprehensive framework and a thorough picture of the global categorization of the CTV isolates and their origin.

## Materials and Methods

### Plant Acquisition and Virus Maintenance

**FIGURE 1:**
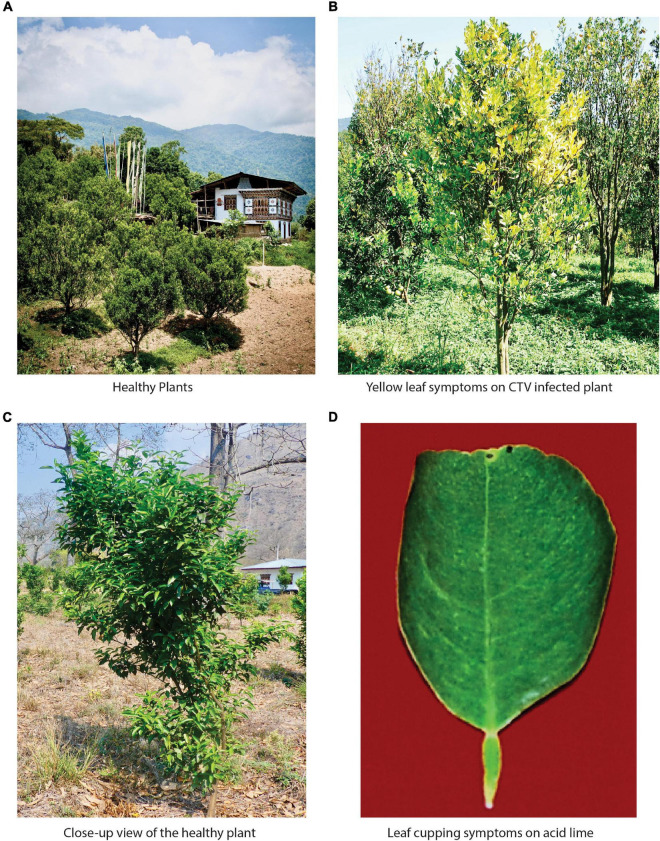
Bhutan represents the richest diversity of tristeza variants found across the world. **(A)** Citrus cultivation scenario in Bhutan. Almost every household grows oranges in their backyard and surround their houses. **(B)** Typical CTV-infected plant in the field showing the yellowing of leaves. **(C)** A closer view of the healthy plant in the field. **(D)** One of the typical symptoms of a severely tristeza-infected plant is leaf cupping on acid lime.

**FIGURE 2:**
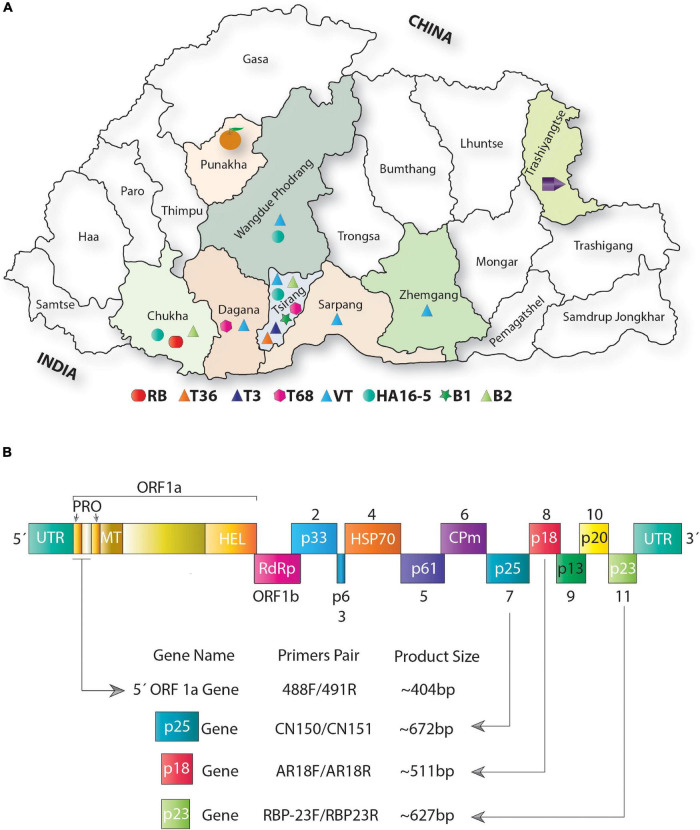
Distribution of citrus tristeza virus (CTV) variants across major citrus growing regions of Bhutan. **(A)** Map showing the geographic locations of Bhutan. Districts filled with color from where the samples were collected. Phylogenetically distinct variants identified among Bhutanese CTV isolates are shown with different colored shapes and placed at the bottom of the map (please refer to Figures 4, 5 for detailed phylogenetic classification of CTV variants). The district “Tsirang” was identified as the hotspot of the CTV variants showing the presence of seven major CTV variants out of nine variants identified in this analysis. The district “Chukha” shows the unique presence of a novel variant RB found absent in other districts of Bhutan. Most districts where samples were collected show a high incidence of CTV. The district where CTV was detected with unknown variants is shown with a purple pentagon cartoon. Districts where no CTV and its variants were detected are indicated by an orange cartoon. **(B)** Schematic representation of the genome organization of CTV: colored boxes depict the complete open reading frame (ORF) of the protein-coding and the untranslated region (UTR). Protein coding genes are labeled from 1 to 11. Four CTV genomic locations were targeted for PCR amplification shown with the help of arrows. Primers pair was used to PCR amplify these four genomic locations, and the size of the PCR-amplified product obtained is indicated at the bottom of the image. PRO, papain-like protease domain; MT, methyltransferase domain; HEL, helicase domain; RdRp, RNA-dependent RNA polymerase protein; HSP, heat shock protein; and CPm, minor capsid protein.

Leaves and twigs from 90 citrus plants suspected of being infected by CTV were collected from different geographical regions of eight (Tsirang, Wangdue Phodrang, Punakha, Trashiyangste, Zhemgang, Dagana, Sarpang, and Chukha) districts of Bhutan (Figures 1, 2A and Table 1). These samples were assayed for CTV using conventional RT-PCR, as reported earlier by Ghosh et al. (2021). We also used the biological indexing technique to test representative samples of each district. This was done by side and wedge grafting in 10–12 months old seedlings of acid lime (*Citrus aurantifolia*) that were maintained in an insect-proof screen house at the Indian Council of Agricultural Research-Central Citrus Research Institute (ICAR-CCRI) Nagpur, India.

**TABLE 1:** Details of samples collected from different geographical regions of Bhutan and presence or absence of citrus tristeza virus (CTV) tested by RT-PCR.

Sr. no	Sample code	Citrus cultivar	Botanical name	Location	Symptoms	Target genomic region of CTV			
						5′ORF 1a	p25	p23	p18
1	Bhu-Ts-1	Local mandarin	*Citrus reticulata*	Tsirang	YL	**+**	**+**	**+**	**+**
2	Bhu-Ts-2	Local mandarin	*Citrus reticulata*	Tsirang	YL, Chl	**+**	**+**	**+**	**+**
3	Bhu-Ts-3	Local mandarin	*Citrus reticulata*	Tsirang	D, YL, PG	**+**	**+**	**+**	**+**
4	Bhu-Ts-4	Local mandarin	*Citrus reticulata*	Tsirang	D	**+**	**+**	**+**	**+**
5	Bhu-Ts-5	Local mandarin	*Citrus reticulata*	Tsirang	YL	**+**	**+**	**+**	**+**
6	Bhu-Ts-6	Local mandarin	*Citrus reticulata*	Tsirang	VC, Chl, St	**+**	**+**	**+**	**+**
7	Bhu-Ts-7	Local mandarin	*Citrus reticulata*	Tsirang	AH	**+**	**+**	**+**	**+**
8	Bhu-Ts-8	Local mandarin	*Citrus reticulata*	Tsirang	D	**+**	**+**	**+**	**+**
9	Bhu-Ts-9	Local mandarin	*Citrus reticulata*	Tsirang	D	**+**	**+**	**+**	**+**
10	Bhu-Ts-10	Local mandarin	*Citrus reticulata*	Tsirang	D	**+**	**+**	**+**	**+**
11	Bhu-Ts-11	Shemjong lime	*Citrus aurantifolia*	Tsirang	PG, Chl	**+**	**+**	**+**	**+**
12	Bhu-Ts-12	Teishuponkan	*Citrus poonensis*	Tsirang	VCc, Chl	**+**	**+**	**+**	**+**
13	Bhu-Ts-13	Tarku	*Citrus reticulata*	Tsirang	VC, VF, Chl	**+**	**+**	**+**	**+**
14	Bhu-Ts-14	Fortunella	*Citrus japonica*	Tsirang	D, St	**+**	**+**	**+**	**+**
15	Bhu-Ts-15	Dorokhamandarian	*Citrus reticulata*	Tsirang	D, VC. VF	**+**	**+**	**+**	**+**
16	Bhu-Ts-16	27/28	*Citrus reticulata*	Tsirang	Chl, St	**+**	**+**	**+**	**+**
17	Bhu-Ts-17	Okitsuwase	*Citrus sinensis*	Tsirang	Chl, D	**+**	**+**	**+**	**+**
18	Bhu-Ts-18	Othaponkan	*Citrus poonensis*	Tsirang	VC, VF, Chl, St	**+**	**+**	**+**	**+**
19	Bhu-Ts-19	Yushidaponkan	*Citrus poonensis*	Tsirang	D, Chl	**+**	**+**	**+**	**+**
20	Bhu-Ts-20	Clementine	*Citrus clementina*	Tsirang	D, VC	**+**	**+**	**+**	**+**
21	Bhu-Ts-21	Local mandarin	*Citrus reticulata*	Tsirang	AH	**−**	**−**	**−**	**−**
22	Bhu-Ts-22	Local mandarin	*Citrus reticulata*	Tsirang	Chl, D, PG	**+**	**+**	**+**	**+**
23	Bhu-Ts-23	Caracara	*Citrus sinensis*	Tsirang	Chl, VC	**+**	**+**	**+**	**+**
24	Bhu-Ts-24	Citron	*Citrus medica*	Tsirang	PG, D, VC	**+**	**+**	**+**	**+**
25	Bhu-Ts-25	Local mandarin	*Citrus reticulata*	Tsirang	AH	**−**	**−**	**−**	**−**
26	Bhu-Ts-26	Local mandarin	*Citrus reticulata*	Tsirang	Chl, D, VC	**+**	**+**	**+**	**+**
27	Bhu-Ts-27	Local mandarin	*Citrus reticulata*	Tsirang	PG, Chl	**+**	**+**	**+**	**+**
28	Bhu-Ts-28	Local mandarin	*Citrus reticulata*	Tsirang	Chl, PG, VC	**+**	**+**	**+**	**+**
29	Bhu-Ts-29	Local mandarin	*Citrus reticulata*	Tsirang	PG, Chl	**+**	**+**	**+**	**+**
30	Bhu-Ts-30	Local mandarin	*Citrus reticulata*	Tsirang	VC, VF, Chl, St	**+**	**+**	**+**	**+**
31	Bhu-Ts-31	Pomelo	*Citrus grandis*	Tsirang	YL	**−**	**−**	**−**	**−**
32	Bhu-Da-32	Local mandarin	*Citrus reticulata*	Dagana	D, YL	**+**	**+**	**+**	**+**
33	Bhu-Da-33	Rangpur lime	*Citrus limonia*	Dagana	AH	**−**	**−**	**−**	**−**
34	Bhu-Da-34	Local mandarin	*Citrus reticulata*	Dagana	D, YL	**+**	**+**	**+**	**+**
35	Bhu-Da-35	Rangpur lime	*Citrus limonia*	Dagana	AH	**−**	**−**	**−**	**−**
36	Bhu-Da-36	Local mandarin	*Citrus reticulata*	Dagana	YL	**+**	**+**	**+**	**+**
37	Bhu-Da-37	Rangpur lime	*Citrus limonia*	Dagana	Chl, YL	**+**	**+**	**+**	**+**
38	Bhu-Da-38	Local mandarin	*Citrus reticulata*	Dagana	YL, Chl	**+**	**+**	**+**	**+**
39	Bhu-Ts-39	Local mandarin	*Citrus reticulata*	Tsirang	AH	**−**	**−**	**−**	**−**
40	Bhu-Ts-40	Hayaka,	*Citrus reticulata*	Tsirang	AH, Chl	**+**	**+**	**+**	**+**
41	Bhu-Ts-41	Berti pomelo	*Citrus grandis*	Tsirang	AH	**−**	**−**	**−**	**−**
42	Bhu-Ts-42	Hayaka	*Citrus reticulata*	Tsirang	D	**+**	**+**	**+**	**+**
43	Bhu-Ts-43	Local T-13	*Citrus reticulata*	Tsirang	AH	**−**	**−**	**−**	**−**
44	Bhu-Ts-44	Clementine	*Citrus reticulata*	Tsirang	YL, D	**+**	**+**	**+**	**+**
45	Bhu-Ts-45	Salustiana	*Citrus sinensis*	Tsirang	VL, St	**+**	**+**	**+**	**+**
46	Bhu-Ts-46	Local mandarin	*Citrus reticulata*	Tsirang	YL	**−**	**−**	**−**	**−**
47	Bhu-Ts-47	Otsu-4	*Citrus reticulata*	Tsirang	YL, VC	**+**	**+**	**+**	**+**
48	Bhu-Ts-48	Ichang papeda	*Citrus ichangensis*	Tsirang	YL, VC, Chl	**+**	**+**	**+**	**+**
49	Bhu-Ts-49	Ryan	*Citrus sinensis*	Tsirang	AH	**−**	**−**	**−**	**−**
50	Bhu-Ts-50	Narng mandarin	*Citrus reticulata*	Tsirang	AH	**−**	**−**	**−**	**−**
51	Bhu-Ts-51	Dagana mandarin	*Citrus reticulata*	Tsirang	VC, Chl	**+**	**+**	**+**	**+**
52	Bhu-Ts-52	Samtse mandarin	*Citrus reticulata*	Tsirang	AH	**−**	**−**	**−**	**−**
53	Bhu-Ts-53	Khengkhar mandarin	*Citrus reticulata*	Tsirang	YL, St, VF	**+**	**+**	**+**	**+**
54	Bhu-Ts-54	Tsirang mandarin	*Citrus reticulata*	Tsirang	VC, VF,	**+**	**+**	**+**	**+**
55	Bhu-Ts-55	Jongkhar mandarin	*Citrus reticulata*	Tsirang	VF, Chl,	**+**	**+**	**+**	**+**
56	Bhu-Ts-56	Shumar mandarin	*Citrus reticulata*	Tsirang	Chl, St	**+**	**+**	**+**	**+**
57	Bhu-Ts-57	Chukha mandarin	*Citrus reticulata*	Tsirang	AH	**−**	**−**	**−**	**−**
58	Bhu-Wa-58	Local mandarin	*Citrus reticulata*	Wangdue Phodrang	AH	**−**	**−**	**−**	**−**
59	Bhu-Wa-59	Pomelo	*Citrus grandis*	Wangdue Phodrang	YL	**−**	**−**	**−**	**−**
60	Bhu-Wa-60	Euraka	*Citrus limon*	Wangdue Phodrang	VC, VF, Chl	**+**	**+**	**+**	**+**
61	Bhu-Wa-61	Grapefruit	*Citrus paradisi*	Wangdue Phodrang	AH	**−**	**−**	**−**	**−**
62	Bhu-Wa-62	Mandarin	*Citrus reticulata*	Wangdue Phodrang	VC, VF,	**+**	**+**	**+**	**+**
63	Bhu-Wa-63	Mandarin	*Citrus reticulata*	Wangdue Phodrang	Chl, PG	**+**	**+**	**+**	**+**
64	Bhu-Pu-64	Local mandarin	*Citrus reticulata*	Punakha	AH	**−**	**−**	**−**	**−**
65	Bhu-Tr-65	Local mandarin	*Citrus reticulata*	Trashiyangtse	AH	**−**	**−**	**−**	**−**
66	Bhu-Tr-66	Local mandarin	*Citrus reticulata*	Trashiyangtse	VC, VF	**+**	**+**	**+**	**+**
67	Bhu-Tr-67	Local mandarin	*Citrus reticulata*	Trashiyangtse	AH	**−**	**−**	**−**	**−**
68	Bhu-Zh-68	Local mandarin	*Citrus reticulata*	Zhemgang	VC, VF, Chl, PG	**+**	**+**	**+**	**+**
69	Bhu-Zh-69	Local mandarin	*Citrus reticulata*	Zhemgang	YL, VC	**+**	**+**	**+**	**+**
70	Bhu-Zh-70	Local mandarin	*Citrus reticulata*	Zhemgang	D, PG	**+**	**+**	**+**	**+**
71	Bhu-Zh-71	Local mandarin	*Citrus reticulata*	Zhemgang	D, St	**+**	**+**	**+**	**+**
72	Bhu-Zh-72	Local mandarin	*Citrus reticulate*	Zhemgang	VC, VF	**+**	**+**	**+**	**+**
73	Bhu-Zh-73	Local mandarin	*Citrus reticulata*	Zhemgang	AH	**−**	**−**	**−**	**−**
74	Bhu-Da-74	Local mandarin	*Citrus reticulata*	Dagana	AH	**−**	**−**	**−**	**−**
75	Bhu-Da-75	Local mandarin	*Citrus reticulata*	Dagana	D, PG	**+**	**+**	**+**	**+**
76	Bhu-Da-76	Local mandarin	*Citrus reticulata*	Dagana	PG, Chl, VC	**+**	**+**	**+**	**+**
77	Bhu-Sa-77	Local mandarin	*Citrus reticulata*	Sarpang	AH	**−**	**−**	**−**	**−**
78	Bhu-Sa-39	Local mandarin	*Citrus reticulata*	Sarpang	D, Chl	**+**	**+**	**+**	**+**
79	Bhu-Sa-79	Local mandarin	*Citrus reticulata*	Sarpang	D, PG	**+**	**+**	**+**	**+**
80	Bhu-Sa-80	Local mandarin	*Citrus reticulata*	Sarpang	VC, VF	**+**	**+**	**+**	**+**
81	Bhu-Sa-81	Local mandarin	*Citrus reticulata*	Sarpang	PG, YL	**+**	**+**	**+**	**+**
82	Bhu-Sa-82	Local mandarin	*Citrus reticulata*	Sarpang	YL, VC	**+**	**+**	**+**	**+**
83	Bhu-Sa-83	Local mandarin	*Citrus reticulata*	Sarpang	AH	**−**	**−**	**−**	**−**
84	Bhu-Sa-84	Local mandarin	*Citrus reticulata*	Sarpang	AH	**−**	**−**	**−**	**−**
85	Bhu-Ch-85	Dorokha mandarin	*Citrus reticulata*	Chukha	Chl, YL	**+**	**+**	**+**	**+**
86	Bhu-Ch-86	Wangkhartshalv-I	*Citrus reticulata*	Chukha	AH	**−**	**−**	**−**	**−**
87	Bhu-Ch-87	Wangkhartshalvngam	*Citrus reticulata*	Chukha	VC, VF, Chl, St	**+**	**+**	**+**	**+**
88	Bhu-Ch-88	Wangkhartshalvngam	*Citrus reticulata*	Chukha	AH	**−**	**−**	**−**	**−**
89	Bhu-Ch-89	Satsuma manadarin	*Citrus reticulata*	Chukha	PG, Chl, D	**+**	**+**	**+**	**+**
90	Bhu-Ch-90	Lemon euraka	*Citrus limon*	Chukha	VC, Chl	**+**	**+**	**+**	**+**
**Total no of positive samples (disease incidence)**						**64 (71.11%)**			

*Chl, Chlorosis; AH, Apparently healthy; D, Declined; YL, Yellow leaves; PG, Poor growth; VC, Vein clearing; VF, Vein flecking; St, Stunting; +, CTV positive sample; –, CTV negative sample.*

### Sample Processing and RNA Extraction

Symptomatic leaves from all collected samples were thoroughly washed with double-distilled water, wiped with 70% ethanol to avoid surface contamination, and blot dried. Midrib portions of the leaves were excised and ground in liquid nitrogen. Approximately 100 mg of ground sample was used for total RNA extraction using the RNeasy Plant Mini Kit (Qiagen, Hilden, Germany) as per the manufacturer’s protocol. The extracted RNA was dissolved into the Tris-EDTA (TE) buffer and stored at −80°C for further analysis. The concentration of total genomic RNA was assessed by a NanoDrop 2000 spectrophotometer (Thermo Fisher Scientific, Delaware, United States), and quality was determined by 2% agarose gel, stained with 0.5 μg/ml of ethidium bromide, and visualized in a Gel documentation system (G: Box, Syngene, Frederick, United States).

### Primer Designing

Primers were designed against the CTV-targeted genomic locations of 5′ORF1a, p25, p23, and p18 using primer 3v.0.4.0 tool.^1^ Primer specificity was then checked using primer BLAST software at National Center for Biotechnology Information (NCBI)^2^ to avoid cross-reaction with other pathogens or targets. The primers were finally custom synthesized from IDT (Integrated DNA Technologies, Coralville, IA, United States) (Table 2).

**TABLE 2:** Details of the primers used in the present study.

Sr. no	Primer code	Sequence	Annealing temp.	Amplicons size	Target genomic regions	References
1	488F 491R	5′ TGTTCCGTCCTGSGCGGAAYAATT 3′ 5′ GTGTARGTCCCRCGCATMGGAACC 3′	58°C	404 bp	5′ ORF 1a	Roy et al., 2005a
2	CN150 CN151	5′ATATATTTACTCTAGATCTACCATGGACGACGAAACAAA 3′ 5′ GAATCGGAACGCGAATTCTCAACGTGTTAAATTTCC 3′	61°C	672 bp	p25	Cevik et al., 1996
3	RBP-23F RBP-23R	5′ ATGAACGATACTAGCGGAC 3′ 5′ GATGAAGTGGTGTTCACGG 3′	52°C	627 bp	p23	Kokane S. B. et al., 2020
4	AR18F AR18R	5′ ATGTCAGGCAGCTTGGGAAATT 3′ 5′ TTCGTGTCTAAGTCRCGCTAAACA 3′	62°C	511 bp	P18	Roy et al., 2005b

### Establishing Citrus Tristeza Virus Culture Confirmation by RT-qPCR and Electron Microscopy

Total RNA extracted from graft-inoculated samples were used for RT-qPCR assay using CTV-specific primer–probe combination (P25-F/p25-R) and corresponding CTV-FAM probe [labeled with 6-carboxy-fluorescein (FAM) reporter dye at the 5′ terminus and the Black Hole Quencher (BHQ)-1 dye at the 3′ terminus]. The TaqMan-qPCR assay for CTV was performed using a StepOne Real-Time PCR System (Applied Biosystems) in two steps as described by Ghosh et al. (2020) with the following conditions: 95°C for 2 min (initial denaturation), followed by 40 cycles at 95°C for 15 s, annealing, and primer extension simultaneously for 1 min at 60°C. All experimental reactions were conducted in triplicate along with non-template controls (NTC), and the data were analyzed using StepOne Software v2.1. Furthermore, the graft-inoculated samples were also tested by electron microscopy in leaf dip preparation as reported by Ghosh et al. (2009).

### Detection of Citrus Tristeza Virus in Field Samples Using Conventional RT-PCR

The total genomic RNA extracted from the leaves of CTV-suspected samples were used to perform RT-PCR in two steps with CTV 5′ORF1a gene-specific primer set, 488F/491R (Roy et al., 2005a; Atallah et al., 2016). In the first step, cDNA was synthesized in a 15 μl reaction volume. The reaction contained 1× first-strand buffer, 0.5 mM deoxyribonucleotide triphosphates (dNTPs) (Promega, Madison, United States), 15.6 U of RNAsin (Promega, Madison, United States), 0.4 μM reverse primer (491R), 6 μl of total RNA, and 120 units of M-MLV reverse transcriptase (Promega, Madison, United States). The reaction was carried out in a thermal cycler (Bio-Rad 100 Thermal Cycler, California, United States) with extension at 42°C for 50 min and denaturation at 72°C for 10 min. In the second step, a 1.75 μl aliquot of cDNA was used as a template in a 25 μl reaction mixture containing 1× PCR buffer, 0.2 μM of each primer (488F/491R), 0.2 mM of the dNTPs mix, 1.5 mM MgCl_2_, and 1.25 U of GoTaq DNA polymerase (Promega, Madison, United States). The amplification was with one cycle of 3 min at 94°C followed by 35 cycles of 0.30 min at 94°C, 0.45 min at 58°C, 1 min at 72°C, and final extension for 10 min at 72°C. The amplified RT-PCR products of the 5′ORF 1a fragment were analyzed on 1.2% agarose gel. Three more genomic regions of CTV, *viz.*, p25, p23, and p18 genes were also used for detection and molecular characterization of CTV isolates. The primer pairs specific for p25 (CN150/CN151) (Cevik et al., 1996), p23 (RBP-23F/RBP-23R) (Kokane S. B. et al., 2020), and p18 (AR18F/AR18R) (Roy et al., 2005b) were used to perform the RT-PCR (Table 2). The amplification program for the p25, p23, and p18 genes were the same as described above with modifications in annealing time and temperature, *i.e.*, 0.45 min at 61°C for p25, 0.40 min at 52°C for p23, and 0.35 min at 62°C for p18 gene. The amplified RT-PCR products were analyzed on 1.2% agarose gel.

### Nucleotide Sequence Analysis of the 5′ and 3′-Terminal Regions of Citrus Tristeza Virus Genome

The amplified products of four genomic regions were excised and eluted from the agarose gel using the GenElute Gel Extraction Kit (Sigma-Aldrich, Bengaluru, India) and sequenced from both ends DNA sequencing facility (Eurofins Genomics, Bengaluru, India). The forward and reverse sequences were assembled into one complete contig of the target gene and eliminated the repeated sequences. To assess the sequence similarity, the prepared contigs were analyzed by the basic local alignment search tool (BLAST) of the NCBI. The confirmed nucleotide sequences were translated using the online software EXPASY translate tool.^3^ Sequence similarities of proteins were identified using the BLASTp. Assembled sequences of each gene were then deposited into GenBank using BankIt-NCBI-NIH software.^4^ Assembled nucleotide sequences of the four genomic regions were further used to analyze genetic variations biostatistically and pair-wise identity using GeneDoc software among the CTV isolates (Nicholas et al., 1997).

### Sequence Retrieval, Extraction of Genomic Location, Sequence Alignment, and Phylogeny Reconstruction

Depending on the amino acids or nucleotide sequences, Blastp, TBlastn, or Blastn searches were performed with the default parameters using 5′ORF1a, p25, p23, and p18 as a query against all GenBank deposited sequences, including the whole-genome tristeza sequences available at the NCBI. Along with the GenBank deposited CTV variants, a total of 53 whole-genome CTV nucleotide sequences were retrieved and downloaded, and local standalone BLAST (Camacho et al., 2009) searches were performed against the retrieved genomic sequences. In local BLAST searches, the amino acid sequences of 5′ORF1a, p23, p18, and p25 were again used as a query against the whole-genomes CTV sequences in Tblastn searches to identify the corresponding amino acid sequences. The whole-genome nucleotide sequence was then translated in three frames at https://www.bioinformatics.org/sms2/trans_map.html, and the nucleotide sequence of the corresponding genomic region encoded by these proteins was extracted manually. Individual DNA and the protein sequences against these four genomic regions extracted for a particular isolate from the whole-genome sequences along with all other retrieved sequences from NCBI are presented in Supplementary Excel File 1 and freely available for download. A novel phylogenetic reconstruction approach was then used in this analysis using the concatenated nucleotides and protein sequences of the four genomic regions of the Bhutanese variants and the GenBank deposited sequences. Due to high sequence similarities at the protein level, the phylogeny was performed using the corresponding DNA sequences to determine the greater sequence variations due to the presence of both synonymous (mutations in the codon that do not change the amino acids) and non-synonymous (mutations that alter the amino acids) changes. Both the amino acid and nucleotide sequences were aligned in MUSCLE (Edgar, 2004), and maximum likelihood (ML) trees were inferred using PhyML v3.0 (Guindon and Gascuel, 2003; Guindon et al., 2010), with the best-fit evolutionary model identified using the Akaike information criterion (AIC) criterion estimated by ProtTest (Abascal et al., 2005). The JTT substitution matrix was used for the amino acid sequences and the GTR substitution model for the nucleotide sequences while estimating the tree topology, branch lengths, amino acid equilibrium frequencies, fraction of invariable sites, and discrete-gamma distributed substitution rates. Clade support was calculated using the SH-like approximate likelihood ratio test (Anisimova et al., 2011). The resulting phylogenetic trees were viewed online and edited with iTol version 2.0 (Letunic and Bork, 2007). The vector graphics file was then imported onto Adobe Illustrator version CS6 for editing and final exporting of the high-resolution picture for publication.

## Results

### Symptomatology, Bioassay, RT-qPCR, and Electron Microscopy

During field surveys in the different districts of Bhutan (Figure 2A), citrus trees showed the typical characteristic of tristeza symptoms, specifically chlorosis, yellow leaves, leaf cupping, vein clearing, vein flecking, declined condition, poor growth, and vigor. Stunting in diverse species was also observed, for instance, in mandarin (*Citrus reticulata*), pomelo (*Citrus grandis*), lime (*Citrus aurantifolia*), citron (*Citrus medica*), and other citrus cultivars or hybrids. However, few citrus trees found seemed to be healthy (Table 1). We performed Koch’s postulate successfully for CTV in acid lime indicator plants. The virus-inoculated plants developed vein clearing, leaf cupping, temporary yellowing, and stunting of young seedlings (Supplementary Figures 1A,B). The titers of CTV in graft-inoculated plants were confirmed by RT-qPCR. However, the virus titer was varied from plant to plant, and Ct (cycle threshold) values were found ranging from 19.25 to 29.12 per 500 ng/μl of RNA extracted (Supplementary Figure 1E). Furthermore, under electron microscopy, CTV particles having the size of 2,000 × 11 nm were also observed (Supplementary Figure 1D).

### RT-PCR Detection and Disease Incidence

**FIGURE 3:**
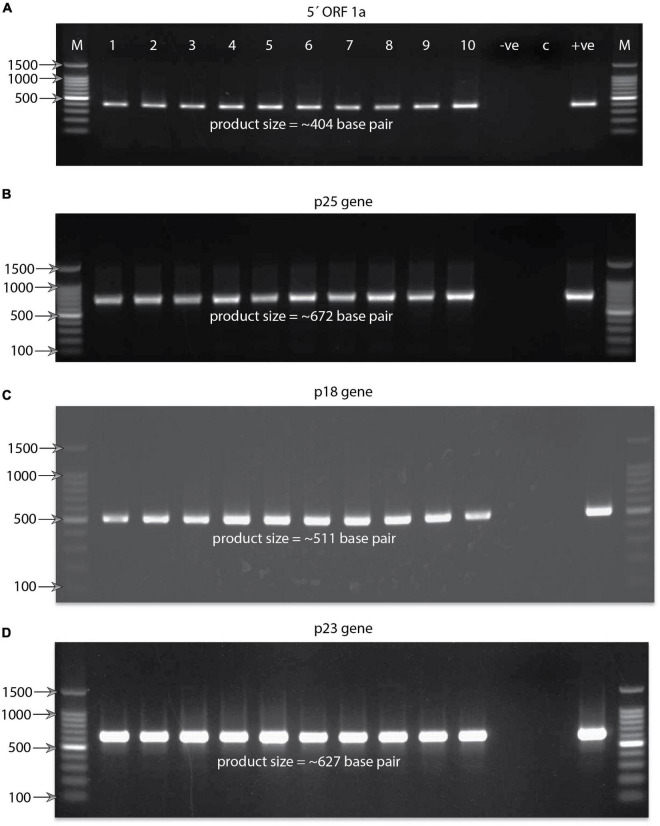
Agarose gel electrophoresis pictures showing RT-PCR-amplified product. **(A)** Product size of (∼404) base pair obtained targeting the 5′ORF1a gene against the Bhutanese CTV variants. At the top of the gel lanes from right to left are labeled as follows: lane M, 100-base-pair DNA ladder; lanes 1–10, representatives of the Bhutanese CTV variants; lane −ve, reaction control; lane C, healthy plant control; and lane + ve, positive control. For clarity, labeling is not shown for the other three gel pictures. The same labeling of the first gel applies to all four gel pictures. Some of the significant DNA ladder sizes are labeled and shown with the help of arrows. **(B)** Gel showing the RT-PCR product size of ∼672 base pairs obtained targeting the p25 coat protein gene of the Bhutanese CTV variants. **(C)** Gel showing the RT-PCR product size of ∼511 base pairs obtained targeted against the p18 gene of the Bhutanese CTV variants. **(D)** Gel showing the RT-PCR product size of ∼627 base pairs targeted against the RNA binding p23 gene of the Bhutanese CTV variants. Primer pairs used to amplify these products are mentioned in Figure 2B and section “Materials and Methods.”

RT-PCR detected the CTV variants in all the collected samples by separate targeted gene-specific primer sets (Figure 2B and Table 2). For example, the 5′ORF1a-specific primers pair 488F/491R targeting the genomic region between 1,082 and 1,484 nucleotides on the CTV genome resulted in an intense band of ∼404 bp. Of the 90 samples collected, 64 were found positive for CTV. These samples also tested positive against p25, p23, and p18 gene-specific primers and showed the expected amplicons of ∼672, ∼627, and ∼511 bp, respectively. Amplicons of 10 representative isolates for each gene were separated on a 1.2% agarose gel (Figures 3A–D). No amplification was observed with either the healthy citrus plant or the non-template control (NTC). The extent of the disease incidence was at a higher level; surprisingly, very few citrus trees were observed to be healthy (Table 1). The average CTV disease incidence was nearly 71.11% (Table 1). The percent of tree infection varied based on citrus cultivars and locations of the orchards. The highest CTV incidence was recorded in the Zhemgang district (83.33%), followed by Tsirang (78%), Dagana (70%), Chukha (66.66%), Sarpang (62.5%), Wangdue Phodrang (50%), and Trashiyangtse (33.33%).

### Sequence Variations and Pair-Wise Identity Among the Citrus Tristeza Virus Isolates

The assembled sequences of four genomic regions of CTV were deposited into GenBank databases under the accession numbers listed in Table 3. Furthermore, these sequences were used to analyze genetic variations and pair-wise identity among all Bhutanese CTV variants. The nucleotide variation in the 5′ORF1a region ranged from 0.0 to 0.18 with an average of 0.06, and the pair-wise nucleotide identities were found to be 84–100% across all CTV variants. Genetic variations in the p25 gene varied from 0.02 to 0.10 with an average of 0.059 and 89–100% sequence identity within isolates. The p23 gene showed 88–100% nucleotide identity, and sequence variations ranged from 0.0 to 0.13 with an average of 0.48, whereas the p18 gene showed 91–100% identity, and nucleotide variation ranged from 0.0 to 0.11 with an average of 0.05.

**TABLE 3:** Citrus tristeza virus (CTV) isolates collected from different geographic regions of Bhutan. Four different specific genomic regions are sequenced and their accession numbers are presented.

Sr. no	Sample code	Concatenated study based CTV groups	CTV accession			
			5′ ORF 1a	p25	p23	p18
1	Bhu-Ts-1	VT-B	SND	SND	MN104226	SND
2	Bhu-Ts-2	VT-B	SND	SND	MN104227	SND
3	Bhu-Ts-3	VT-B	SND	SND	MN104228	MN117985
4	Bhu-Ts-4	VT-B	SND	MN104221*	MN104229	MN117986
5	Bhu-Ts-5	VT-B	SND	SND	MN104230	MN117987
6	Bhu-Ts-6	VT-B	SND	MN104222*	MN117969	SND
7	Bhu-Sa-7	VT-B	MN384882	SND	SND	SND
8	Bhu-Ts-8	VT-B	SND	MN104223*	MN117970	MN117988
9	Bhu-Ts-9	VT-B	SND	MN104224*	MN117971	MN117989
10	Bhu-Ts-10	VT-B	SND	MN104225*	MN117972	MN117990
11	Bhu-Ts-11	B2	MN384885	SND	MN549939	MN580428
12	Bhu-Ts-12	T3	SND	MN366299	MN549947	SND
13	Bhu-Ts-13	T3	MN384883	MN366307	MN549941	MN580427
14	Bhu-Ts-14	T36	SND	MN366297	SND	MN580434
15	Bhu-Ts-15	HA16-5	MN384884	MN366302	MN549942	MN580429
16	Bhu-Ts-16	B2	SND	SND	MN549940	MN580430
17	Bhu-Ts-17	B1	MN651084	SND	MN549944	MN580432
18	Bhu-Ts-18	T3	MN651083	MN366301*	MN549948	MN580435
19	Bhu-Ts-19	VT-B	MN651088	MN366303*	MN549945	MN580436
20	Bhu-Ts-20	T3	MN651089	MN366300*	MN549951	MN580422
21	Bhu-Ts-22	B1	MN651087	MN366306	MN549954	MN580426
22	Bhu-Ts-23	B1	SND	SND	MN549949	MN580433
23	Bhu-Ts-24	B2	MN651085	SND	MN549953	MN580424
24	Bhu-Ts-26	B1	SND	SND	MN549952	MN580423
25	Bhu-Ts-27	T68	MN651086	MN366298	MN549950	MN580437
26	Bhu-Ts-28	VT-B	SND	SND	SND	MN580425
27	Bhu-Ts-29	B2	SND	MN366305	MN549943	MN580431
28	Bhu-Ts-30	VT-B	MN384880	MN366304	MN549946	MN580438
29	Bhu-Da-36	T68	SND	MN101752*	MN137882	MN137877
30	Bhu-Da-38	VT-B	SND	SND	MN137883	MN137878
31	Bhu-Wa-60	VT-B	MN651093	MN366309	MN398270	SND
32	BhuWa-62	HA16-5	MN651091	MN366310	SND	SND
33	Bhu-Zh-68	VT-B	MN651096	MN366311*	MN398271	SND
34	Bhu-Zh-71	VT-B	SND	MN101753*	MN137884	MN137879
35	Bhu-Zh-72	VT-B	MN651094	MN366316	MN398272	SND
36	Bhu-Da-76	VT-B	MN651097	MN366312*	MN398273	MN580439
37	Bhu-Sa-39	VT-B	SND	SND	MN137885	SND
38	Bhu-Sa-79	VT-B	SND	SND	MN137887	MN137881
39	Bhu-Sa-80	VT-B	MN651092	MN366313	MN398274	MN580440
40	Bhu-Sa-81	VT-B	MN651095	SND	SND	MN580441
41	Bhu-Sa-82	VT-B	SND	SND	MN137886	SND
42	Bhu-Ch-87	HA16-5	MN651090	MN366314	MN398277	MN580442
43	Bhu-Ch-89	B1	MN651098	MN366315	MN398278	SND
44	Bhu-Ch-90	RB	SND	MN366317	MN398279	SND
**Major CTV strains**						
45	T36	T 36	U16304	U16304	U16304	U16304
46	T30	T 30	AF260651	AF260651	AF260651	AF260651
47	VT	VT	EU937519	EU937519	EU937519	EU937519
48	T3	T 3	KC525952	KC525952	KC525952	KC525952
49	T68	T 68	JQ965169	JQ965169	JQ965169	JQ965169
50	RB	RB	FJ525434	FJ525434	FJ525434	FJ525434
51	HA16-5	HA16-5	GQ454870	GQ454870	GQ454870	GQ454870

CTV, Citrus tristeza virus; *, Our earlier studied samples; SND, Sequencing not done.

### Molecular Characterization of Citrus Tristeza Virus Variants

**FIGURE 4:**
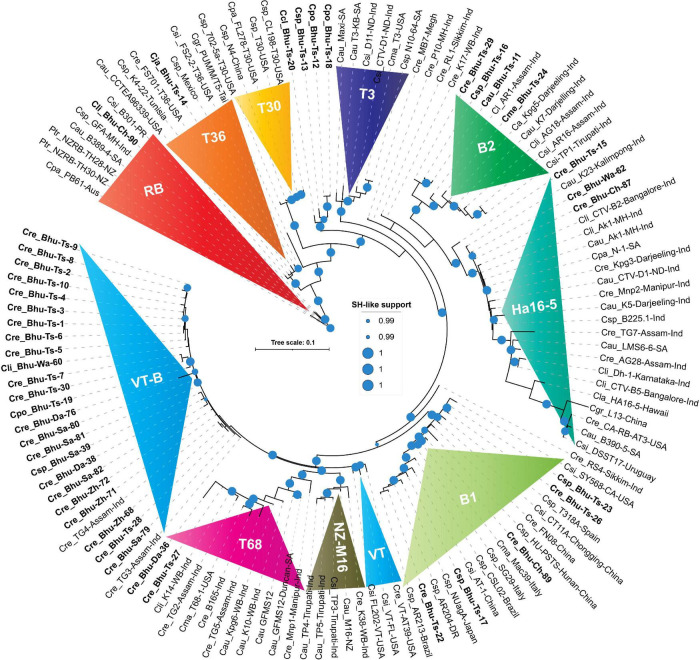
Phylogenetic trees reconstructed using concatenated DNA correspond to four genomic locations showing unprecedented diversities among Bhutanese CTV variants. Maximum likelihood (ML) tree was reconstructed using PHYML3.2.2 (Guindon et al., 2010) with default parameters, except for the model parameter that was set to GTR, and both NNI (nearest-neighbor interchange) and SPR (sub-tree pruning and re-grafting) moves were adopted for their accuracy of branch placement along the tree (Hordijk and Gascuel, 2005). SH-like (Anisimova et al., 2011) support, which is known to outperform bootstrap support (Simmons and Norton, 2014), was used for branch stability. SH-like support of 99% or higher is shown with a cyan color circle. CTV strains were earlier classified under seven (T30, T36, VT, T3, T68, RB, and HA16-5) subtypes or variants (Harper, 2013). In contrast, our concatenated nucleotides-based ML tree showing an additional two (B1 and B2) clades could be confidently allotted with a high SH-like support value. Clades B1 and B2 are named after the Bhutanese variants. Labeling the ML tree was made clearer by using the first letter of the genus and two letters of the citrus species, followed by the isolate or genotype name, place, and the country where it was reported. For clarity, Bhutanese isolates are shown in bold font. For a complete list of the GenBank accessions, species name, isolate or variants name, country name, protein-coding region, and their respective DNA used to build this tree refer to Supplementary Excel File 1, and for the complete list of Bhutanese isolates, refer to Tables 1, 3.

**FIGURE 5:**
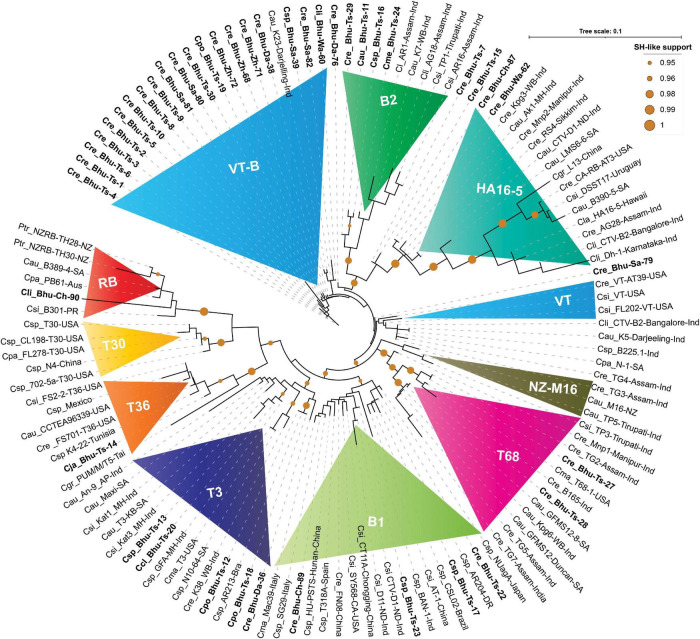
Phylogenetic tree reconstructed using concatenated protein sequences congruent with the nucleotide tree (Figure 4). The maximum likelihood (ML) tree was reconstructed using PHYML3.2.2 (Guindon et al., 2010) with default parameters except for the model parameter that was set to JTT (Jones et al., 1992), and both NNI (nearest-neighbor interchange) and SPR (sub-tree pruning and re-grafting) moves were adopted for better accuracy (Hordijk and Gascuel, 2005). SH-like (Anisimova et al., 2011) support, which is known to outperform bootstrap support (Simmons and Norton, 2014), was used for branch stability. SH-like support of 95% or higher is shown with a mustard color circle. CTV strains were earlier classified under seven (T30, T36, VT, T3, T68, RB and HA16-5) subtypes or variants (Harper, 2013). In contrast, our concatenated protein-based ML tree shows an additional three (B1, B2, and NZ-M16) clades. Clades B1 and B2 are named after the Bhutanese variants. Labeling in the ML tree was made clearer by using the first letter of the genus and two letters of the citrus species, followed by the isolate or genotype name, place, and the country where it was reported. For clarity, Bhutanese isolates are shown in bold font. For a complete list of the GenBank accessions, species name, isolate or variants name, country name, and protein-coding amino acids used to build this tree, refer to Supplementary Excel File 1, and for the complete list of Bhutanese isolates, refer to Tables 1, 3.

Both the concatenated nucleotide and amino-acid-based maximum likelihood (ML) trees are presented in Figures 4, 5. Worldwide CTV isolates have been classified under seven internationally recognized strains (Harper, 2013). However, the ML tree based on both the nucleotides and protein sequences in our analysis identified two (B1 and B2) additional isolates or variants (Figures 4, 5). These trees show remarkable unity in their branching pattern and relationship with neighboring clades. Based on our analysis, Bhutanese and the worldwide CTV isolates could be robustly classified under the following variants described below:

#### Resistance-Breaking Isolate

RB isolate was named after discovering the founding member, *Poncirus trifoliate* resistance-breaking (RB) strain from New Zealand and shown to have 90% nucleotide sequence identity against the neighboring clade T36 (Harper et al., 2010). The presence of an RB strain among the Puerto Rican isolates was also reported (Roy et al., 2013). The RB isolate has not been reported elsewhere from the world, including the Asian subcontinent. Besides establishing for the first time the RB strain from South Africa (B389-4) and Australia (PB61), based on our comparative bioinformatic analysis, here, we report for the first time the presence of RB strain among Bhutanese isolates (Figure 4). Validation in the glasshouse using the host assay system is subject to future further analysis. Our analysis also suggests that a small invariable pentapeptide signature motif “RVENV” is present at the amino terminus of the p23 protein sequence, which separates RB strains from the rest of the CTV isolates worldwide. The Bhutanese variant Bhu-Ch-90 carry these invariable amino acids in its p23 gene and confirms its classification under the RB clade. An Indian isolate (GFA-MH) with only the coat-protein (p25) gene sequence from Maharashtra falls under the RB clade in the nucleotide-based tree (Figure 4) and is segregated under the T3 clade in the amino acid-based tree (Figure 5). Together, these results would suggest the widespread presence of the RB variant than earlier realized poignantly located at the center of origin of citrus.

#### T36 Isolate

T36 isolate was named after the founding member of the Florida decline isolate for which the whole-genome sequence was published as early as 1995 (Karasev et al., 1995), and the whole-genome sequence of T36 from Turkey was revealed recently (Cevik et al., 2013). After analyzing the GenBank deposited sequences, we observed that the T36 strain is widespread in Taiwan, Mexico, Tunisia, and Italy (Figures 4, 5). However, the presence of the T36 isolate has not been reported elsewhere, particularly from the Asian subcontinent, including north-eastern India, which has been considered the likely place of origin of citrus (Wu et al., 2018). For the first time in this analysis, we report and establish the presence of a T36 strain (Bhu-Ts-14) among Bhutanese CTV isolates, which form a strong clade along with other T36 strains (Figures 4, 5).

#### T30 Isolate

This strain was named after Florida isolate T30 (Albiach-Martí et al., 2000). The whole-genome sequence, including the two other isolates from Florida and China, is available in the GenBank (Albiach-Martí et al., 2000). These T30 isolates form a distinct clade in nucleotide and protein-based ML trees (Figures 4, 5). We were unable to identify any Bhutanese isolates that belonged to this clade. However, Bhutanese isolates (Bhu-Ts-12, 13, 18, and 20) form a distinct clade with solid statistical support within the T30 and T3 clade (Figure 4), and it could be the transitional state from which T30 or the T3 sequences might have evolved. However, these Bhutanese isolates come under the T3 clade when the amino-acids-based ML tree was generated (Figure 5). However, our finding of a Chinese isolate that belonged to the T30 group would, for the first time, establish its Asian origin (Figures 4, 5). Besides, the robust sister clade relationship among RB, T30, and T36 with solid statistical support indicated that these isolates might have originated from their ancestral sequences that subsequently gave rise to these three clade members.

#### T3 Isolate

The T3 isolate was named after the founding member recovered from a lime tree in Florida, for which the whole-genome sequence is available in the GenBank. A strain from New Zealand (NZ-M16) has been classified as T3-like, for which the whole-genome sequence is available (Harper et al., 2009). This strain, in our analysis, forms its clade next to the T68 clade with robust statistical support together with other sequences from India that suggested its Asian origin (Figures 4, 5). We retain the name of this clade as NZ-M16 to distinguish it from other CTV isolates (Figures 4, 5). In addition, the GenBank deposited sequences analysis identified several T3 isolates from South Africa: Maxi, T3-KB, N10-64 (Figures 4, 5 and Supplementary Excel File 1). We also identified a T3 isolate from Brazil (GenBank Acc. DQ363400), for which only 5′ORF1a sequence data are available in the GenBank. Several Indian sequences (An-9, Kat1, Kat3, and K38) also fall within this clade with strong statistical support value. Bhutanese sequences, specifically Bhu-Ts-12, 13, 18, and 20, form a strong cluster within the T3 clade based on their amino acid sequences. These results suggest that the T3 isolate is being distributed worldwide, and its origin could be traced to the center of origin of citrus.

#### T68 Isolate

T68 belongs to the Florida isolates for which the whole-genome sequence is available in the GenBank. In addition, the full-genome sequences of two T68 strains that differ in their stem-pitting severity reported from South Africa are also published and available in the GenBank (Cook et al., 2020, 2021). The complete genome sequence of an orange stem-pitting isolate (B165) from India (Roy and Brlansky, 2010) was classified under VT, but our analysis suggested reconsidering it to be classified under T68 isolate (Figures 4, 5). Several isolates, specifically TG2, 3, and 5 from the north-eastern region of India from Assam, form a strong cluster within the T68 clade along with K10, K14, and Kpg6 isolates from Darjeeling, West Bengal, India. The Bhutanese isolate Bhu-Ts-27 based on sequences of all four independent genomic locations formed a strong association within the T68 clade in both nucleotide and protein trees (Figures 4, 5). However, Bhu-Ts-28 based only on the p18 genomic region was found to switch clades between T68 and VT-B. Therefore, we conclude that the Bhu-Ts-28 strain remained unclassified, and the additional genomic sequence would be required for confident classification. Together, our analysis suggests that T68 is distributed worldwide, possibly originating in the north-eastern region of India and Bhutan. We also observed that NZ-M16 formed a sister clade with T68 clade with strong statistical support, which indicated its Indian origin.

#### VT Isolate

The VT strain was named after discovering its founding member from Israel, for which the full-genome sequence is available. In addition to this VT isolate, two other VT strains have been reported from Florida, and all these three VT isolates form an independent clade in the nucleotide tree adjacent to the T68, NZ-M16 clade (Figure 4). Bhutan’s unidentified VT-like sequences get segregated within the same clade (Figure 4). We have to refer to these Bhutanese VT-like sequences as an independent VT-B clade where the B is derived from Bhutan. In the protein tree, some of the sequences from India fall in the same clade as the Florida VT strains without significant statistical support (Figure 5). These Indian sequences, however, form a strong clade together with HA16-5 (Hawaii isolates of CTV), indicating that Florida VT strains are related and originated from any of these four ancestral clades, namely, T68, NZ-M16, VT-B, and HA16-5. In addition, our analyses also have suggested that the ancestry of these four clade members can be traced back to their roots in the north-eastern region of India and Bhutan (Wu et al., 2018).

#### HA16-5 Isolate

This isolate was named after the founding member from Hawaii and was classified as a new genotype for which the whole-genome sequence has been published (Melzer et al., 2010). The CTV isolate LMS6-6 from South Africa was recently classified under HA16-5 clade (Cook et al., 2016). Both LMS6-6 and HA16-5 in our analysis fall in the HA16-5 clade in both nucleotide and protein trees with firm statistical support (Figures 4, 5). Four sequences are supposedly *Poncirus-*resistant breaking strains, namely, CA-RB-AT3 from California, United States (Yokomi et al., 2017), L13 from China (Wang et al., 2019), DSST17 from Uruguay (Benitez-Galeano et al., 2018), and B390-5 from South Africa (Cook et al., 2016) and have been classified under RB clade, which, in our analysis with both nucleotide and protein tree, form a strong clade together with HA16-5 and LMS6-6 (Figures 4, 5). The clade HA16-5 also accommodates several isolates from the southern part of India and the north-eastern region, including Assam and Darjeeling, together with at least three isolates from Bhutan (Bhu-Ts-15, Bhu-Wa-62, and Bhu-Ch-87) (Figures 4, 5). This result suggests that the HA16-5 strains have been distributed worldwide, with the root traced back to Bhutan and Northeast India.

#### B1 Isolate

A severe stem-pitting (SP) isolate from California (SY568) reported to have sequence similarities with Florida and Israel VT strain has been published (Yang et al., 1999). In addition, the whole-genome sequences of two Italian isolates Mac39 and SG29 sequences are also available in the GenBank, of which SG29 was shown to have clustered within the VT-Asian subtypes (Licciardello et al., 2015). Similarly, the whole-genome sequences of severe stem-pitting (SP) isolates from Spain (Ruiz-Ruiz et al., 2006) and Brazilian isolate CSL02 have been classified under the VT group (Matsumura et al., 2017). The full-length genome of NUagA isolate from Japan, which causes seedling yellows, and sequences of four unclassified isolates, namely, HU-PSTS, FN08, CT11A, and AT-1, from China are available in the GenBank. Together with the isolates from India and Bhutanese variants, all these isolates are mentioned here. Specifically, Bhu-Ts-17, Bhu-Ts-22, Bhu-Ts-23, Bhu-Ts-26, Bhu-Ch-89 form an unrelated cluster distinctly different from VT isolates in both nucleotide and protein trees (Figures 4, 5). This result suggests that these isolates should be classified under distinct clade, which we prefer to name B1 clade after Bhutan. Thus, the B1 clade harboring the severe stem-pitting isolates has a worldwide distribution, and its root could be traced back to the north-eastern region of India and Bhutan (Wu et al., 2018).

#### B2 Isolate

B2 isolates form a distinctly different clade in the ML tree with solid statistical support next to the HA16-5 clade in nucleotide and amino acid tree (Figures 4, 5). Isolates of this clade have so far been found only among Bhutanese variants (Bhu-Ts-11, Bhu-Ts-16, Bhu-Ts-24, Bhu-Ts-29) and variants from the north-eastern region of Southeast Asia, including Assam and Darjeeling.

### Distribution of Citrus Tristeza Virus Variants in Bhutan

Phylogenetic analysis using the four genomic locations has allowed us to classify the CTV isolates into nine major groups (RB, T36, T30, T3, T68, VT or VT-B, HA16-5, B1, and B2). Except for the T30 group, Bhutanese isolates have representations in all eight groups indicating greater diversity. Except for the resistant-breaking strain RB, all other seven variants were observed mainly in the Tsirang district. The devastating VT-B strain occurs throughout Bhutan’s major citrus growing districts (Figure 2A). The second most widely distributed variant was HA16-5, which, besides Tsirang, was found in two other regions, Wangdue Phodrang and Chukha districts, and the resistant-breaking RB strain was reported exclusively from the Chukha region (Figure 2A).

## Discussion

CTV, the largest and most complex member of the family *Closteroviridae*, is a phloem-limited virus that infects citrus and closely related species and produces a wide range of characteristic symptoms. Viruses having RNA as their genome have the potential for genetic variations due to their error-prone replication mechanism (Rubio et al., 2001). Similarly, CTV can evolve and exhibit variable pathogenesis on the citrus hosts. Several genomic regions have been targeted and characterized to determine the genetic diversity among the CTV isolates (Ghosh et al., 2008, 2021; Martín et al., 2009; Warghane et al., 2020). It was reported that the 3′ half of the CTV genome, among various isolates, is more conserved (90% identity), while most genetic diversity (44–88% identity) is found at the 5′ terminal half (Atallah et al., 2016). The genetic diversity based on the 5′ terminal region and based on the two combined regions (5′ORF1a and p25 genes) has been reported to discriminate CTV isolates from the northeastern and southern parts of India (Roy et al., 2005a; Tarafdar et al., 2013). In the present study, efforts have been carried out to determine the genetic diversity of CTV isolates from Bhutan using 5′ end ORF1a and three other potential genes of 3′ end, *viz.*, p25, p23, and p18.

In the present investigation, a novel approach of concatenating-independent genomic locations utilizing both the nucleotide and their corresponding amino acid sequences for differentiation of tristeza variants from Bhutan and across the World has been used. This work provides a new framework for revisiting and re-classifying the existing tristeza variants in future studies. The four genomic locations used in this study were in the viral homologous recombination-free regions in the tristeza genome (Vives et al., 2005) and, thus, allowed us to build a robust phylogenetic relationship among global CTV isolates. Although consensus trees generated are robust, common sources of phylogenetic error such as long-branch attraction might skulk; we remain cautious of our interpretation while classifying the new clade such as B2, which is based solely on the 5′ORF1a sequence available at this moment for phylogenetic reconstruction. Despite limited sequence information available in some instances (used for phylogenetic reconstruction), an astounding congruence was noticed in the placement of the isolates within the clade and their sister–clade relationship in the concatenated nucleotide and the protein tree, which further validate the robustness of our classification.

The most striking feature of the results is the sequence diversity assessment among all the Bhutanese CTV variants and establishing their one-to-one relationship with existing worldwide-recognized isolates. Sequences were extracted from the whole-genome sequences and partially sequenced CTV isolates that were previously reported (Hilf et al., 2005; Roy et al., 2005a; Harper, 2013; Ghosh et al., 2021). An exhaustive search was done both for the whole-genome sequence information and the partial sequences available in the GenBank. It was beyond the scope to incorporate all sequences from the GenBank. However, we incorporated all worldwide-recognized CTV isolates that belonged to the seven well-known clades, namely, RB, T36, T30, T3, T68, VT, and HA16-5, and other full-genome and partial CTV isolate sequences supporting the clades. Concatenated nucleotide and protein-based trees are novel approaches that never have been used for plant virus isolates differentiation. We see some strains, such as Bhu-Ts-7, Bhu-Ts-28, and GFA-MH-Ind, switching the places between the clades, mainly because of very limited sequence information available for them. For example, out of four genomic locations targeted in this analysis, sequence information for only one genomic location was available for these isolates for phylogenetic reconstruction. More genomic information for these strains would be required to establish a stable phylogenetic relationship among these Bhutanese isolates. Another most striking result that appeared from our result is placing the roots of these nine clades identified in this analysis to the center of origin of citrus to the north-eastern region of India and Bhutan, which was not defined so far in earlier studies.

Recently, the association of CTV and *Candidatus* Liberibacter asiaticus with citrus decline has been recorded in Bhutan with higher incidence up to 70.58 and 27.45%, respectively (Ghosh et al., 2021). The present investigation also suggests an average incidence of 71.11% (64 out of 90 samples tested positive) of CTV occurring in Bhutan based on targeted genes (5′ORF1a, p25, p23, and p18) by RT-PCR test from eight different districts of Bhutan. The highest CTV incidence was recorded in Zhemgang and Tsirang followed by other districts. We also observed that most of the citrus orchards were neglected, and infestation of aphids was common in most of the surveyed orchards. The tristeza disease is a major threat to the citrus in the northeast region of India, including Bhutan (Borah et al., 2014; Warghane et al., 2020), and aphids may be the major source of virus spread. The evidence generated in the present study will be helpful in quarantine applications in Bhutan. Furthermore, sanitation and the use of virus-free propagation material will be the most powerful method to put the citrus industry of Bhutan on sound scientific footings for increased citrus productivity.

## Data Availability Statement

The datasets presented in this study can be found in online repositories. The names of the repository/repositories and accession number(s) can be found in the article/Supplementary Material.

## Author Contributions

DG, AK, and SK designed the study and developed the methods. DG, KM, SK, and AK prepared the data. All authors analyzed the results and wrote the manuscript.

## Conflict of Interest

The authors declare that the research was conducted in the absence of any commercial or financial relationships that could be construed as a potential conflict of interest.

## Publisher’s Note

All claims expressed in this article are solely those of the authors and do not necessarily represent those of their affiliated organizations, or those of the publisher, the editors and the reviewers. Any product that may be evaluated in this article, or claim that may be made by its manufacturer, is not guaranteed or endorsed by the publisher.

## Footnotes

1
http://bioinfo.ut.ee/primer3-0.4.0/

2
https://www.ncbi.nlm.nih.gov/tools/primer-blast/

3
http://web.expasy.org/translate/

4
https://www.ncbi.nlm.nih.gov/WebSub/
