# Supplementary material for: A Comprehensive Analysis of Citrus Tristeza Variants of Bhutan and Across the World
Source: Front Microbiol. 2022 Apr 8;13:797463. doi: 10.3389/fmicb.2022.797463 (PMC9024366; doi:10.3389/fmicb.2022.797463)
Supplement: Supplementary file 1 [file Data_Sheet_1.docx]

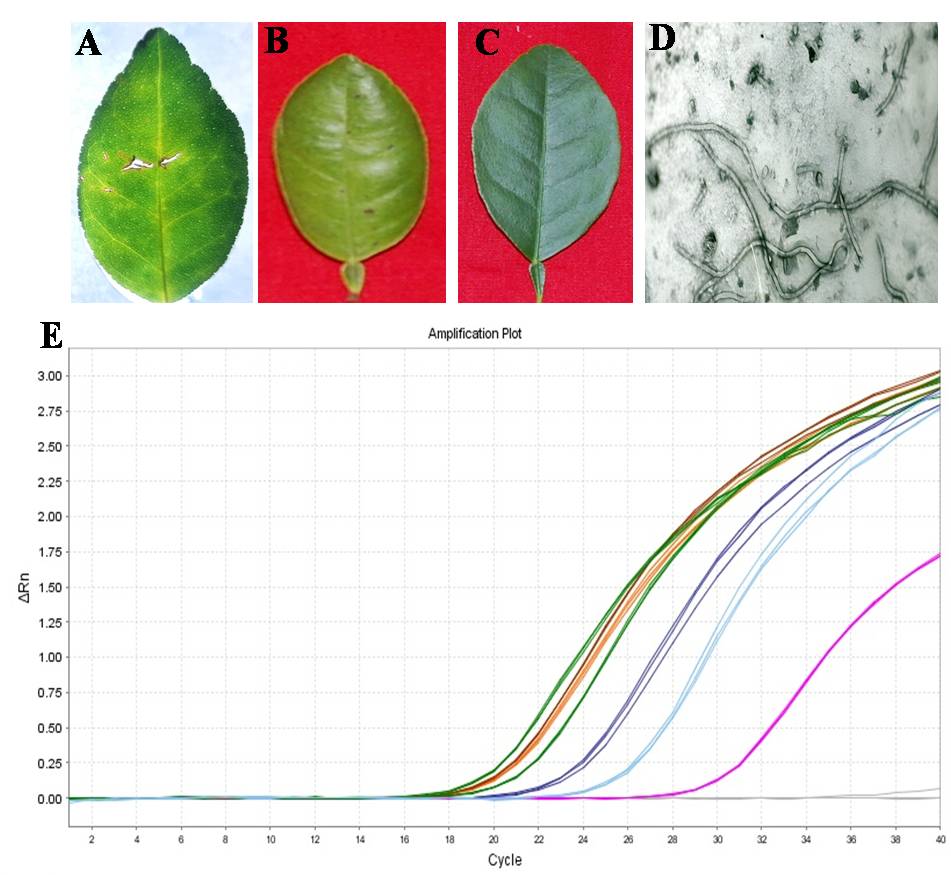


**Supplementary Figure 1**: **(A-B)** Citrus tristeza virus (CTV) induced vein clearing and leaf cupping on acid lime **(C)** Healthy leaf. **(D)** Electron microscopy showing 2000 x 11nm particles of CTV. **(E)** CTV culture confirmation by RT-qPCR assay with P25F/R-CTV FAM primer pair probe using cDNA as template: Amplification plot for representative virus inoculated plants showing average Ct (cycle threshold) values ranging from 19.25 to 29.12, no fluorescence signal was observed with non-template controls (NTC).
